# A random spatial sampling method in a rural developing nation

**DOI:** 10.1186/1471-2458-14-338

**Published:** 2014-04-10

**Authors:** Michelle C Kondo, Kent DW Bream, Frances K Barg, Charles C Branas

**Affiliations:** 1 United States Department of Agriculture—Forest Service, Northern Research Station, 100 North 20th St Suite 205, Philadelphia, PA 19103, USA; 2 Department of Biostatistics and Epidemiology, Perelman School of Medicine, University of Pennsylvania, 423 Guardian Drive, Blockley Hall, Philadelphia, PA 19104-6021, USA; 3 Department of Family Medicine and Community Health, Perelman School of Medicine, University of Pennsylvania, 141-2 Anatomy and Chemistry, 3620 Hamilton Walk, Philadelphia, PA 19104, USA

**Keywords:** Geographic random sampling, Social surveys, Geographic information system (GIS), Global positioning system (GPS), Satellite imagery, Guatemala

## Abstract

**Background:**

Nonrandom sampling of populations in developing nations has limitations and can inaccurately estimate health phenomena, especially among hard-to-reach populations such as rural residents. However, random sampling of rural populations in developing nations can be challenged by incomplete enumeration of the base population.

**Methods:**

We describe a stratified random sampling method using geographical information system (GIS) software and global positioning system (GPS) technology for application in a health survey in a rural region of Guatemala, as well as a qualitative study of the enumeration process.

**Results:**

This method offers an alternative sampling technique that could reduce opportunities for bias in household selection compared to cluster methods. However, its use is subject to issues surrounding survey preparation, technological limitations and in-the-field household selection. Application of this method in remote areas will raise challenges surrounding the boundary delineation process, use and translation of satellite imagery between GIS and GPS, and household selection at each survey point in varying field conditions. This method favors household selection in denser urban areas and in new residential developments.

**Conclusions:**

Random spatial sampling methodology can be used to survey a random sample of population in a remote region of a developing nation. Although this method should be further validated and compared with more established methods to determine its utility in social survey applications, it shows promise for use in developing nations with resource-challenged environments where detailed geographic and human census data are less available.

## Background

Randomly sampled household surveys are necessary tools throughout the world to assess public health status. Geographically-based sampling is often a necessary component of random-sample surveys [1]. Governmental and non-governmental agencies as well as research groups have used such surveys to assess disease burden, vaccination coverage, health-related attitudes or beliefs, and access or barriers to health services among other health indicators. Yet it is rarely feasible to conduct a comprehensive door-to-door survey of every population unit (such as individuals or households), especially in large city- or nation-wide studies. In these cases, researchers must seek to obtain a representative sample, with the random probability sample serving as the gold standard [1]. When all eligible respondents and households are known in a specific target geography, they can be enumerated and then randomly selected to construct a sampling frame [1].

However in many resource-challenged settings, up-to-date and accurate geographic or census data, including maps and household or address lists, are not available [2]. In remote areas, streets and household clusters may be informal, irregular or unnamed and houses may be unnumbered [3]. In humanitarian or post-disaster settings, households may be displaced, and infrastructure interrupted or nonexistent [4].

For social surveys, cluster sampling is a common tool to obtain a representative sample while meeting resource constraints. Researchers have commonly used the Expanded Program on Immunization (EPI) cluster survey method [5], or some variant. In single-stage sampling, the researcher randomly selects clusters and surveys every individual, member or household in that cluster. More commonly, two-stage sampling is used to randomly select individuals or households within a random selection of clusters. GIS/GPS combinations have been used in a two-stage cluster sampling approach to generate the first or starting point, from which surveyors sample along a transect or by proximity [6,7]. GIS/GPS can also be used to locate a single point within a cluster as the survey location [8].

This method has been found to bias sampling methodology for multiple reasons. Cluster sampling can lead to underrepresentation in regions with highly heterogeneous populations or development patterns. Clustering effects are often unaccounted for in analyses. Most criticisms focus on the potential for selection bias, where selection of households in more dense areas and of households near the center of the cluster or the starting point is favored [9-11].

Simple random spatial sampling can be effective in cases such as the one described in this study, where the size of target population and geographic extents do not justify clustering. This method, first proposed by Berry and Baker [12], can be applied with or without clustering and has traditionally been used in ecological surveys [13,14] or studies of air pollution [15] where it is impractical or infeasible to construct or obtain a sampling frame of existing plants, soils or animal species of interest. Spatial sampling methods have also been used in social survey applications [4,16-19]. After the sampling frame is developed, surveyors locate sample locations in the field, such as with a GPS unit or satellite photos [4,18,19]. When population data are completely absent, such as in post-disaster settings or areas were data are out-dated or unrelaiable, advanced enumeration efforts may be necessary [18].

Few variations of this cluster sampling method for application in geographic sub-areas are described in the literature to date. Researchers require a broader set of sampling methods that are appropriate given location conditions, that overcome biases associated with traditional methods, and that are otherwise statistically relevant. In this paper we describe an alternative method, based on a simple stratified random sampling design, for use in areas where clustering is not necessarily required, where street names and numbers are lacking and when comprehensive census data or household/address lists are not available, involving the use of geographic information systems (GIS), global positioning systems (GPS), and satellite imagery [20]. This method introduces opportunities for more efficient sampling and reduced potential for bias in selecting starting points compared to cluster designs. In the analysis we use surveyor feedback to explore issues surrounding survey preparation, technological limitations and in-the-field household selection.

## Methods

### Santiago Atitlán and the Guatemala Health Initiative

This study was conducted in the city of Santiago Atitlán, which lies in the Western Highlands of Guatemala, approximately 3 hours by car to the west of Guatemala City. According to the most recent census conducted by the municipalidad (in 2010), Santiago Atitlán has a total population of 42,594 persons [21]. It is one of approximately a dozen towns on Lake Atitlan, which is set in the vicinity of three volcanoes (one active). Most of these towns, including Santiago Atitlán, are closely nestled to the shoreline, as slopes quickly increase with distance from the lakeshore. Both residential and agricultural (primarily beans and coffee) uses compete for space in low-lying areas near the shoreline. Earthquakes and hurricanes (and subsequent mudslides on steep mountainous slopes) pose significant risk to the people and settlements of this area.

Ninety-four percent of residents are indigenous Tz’utujil Maya [22]. The majority of the population of Santiago Atitlán earns less than the daily Guatemalan minimum wage of approximately $7US [22]. A 2005 health survey found that educations levels are low; approximately 70% of the population has no formal education. Security concerns in some areas are high, mostly in the form of armed thefts.

**Figure 1:**
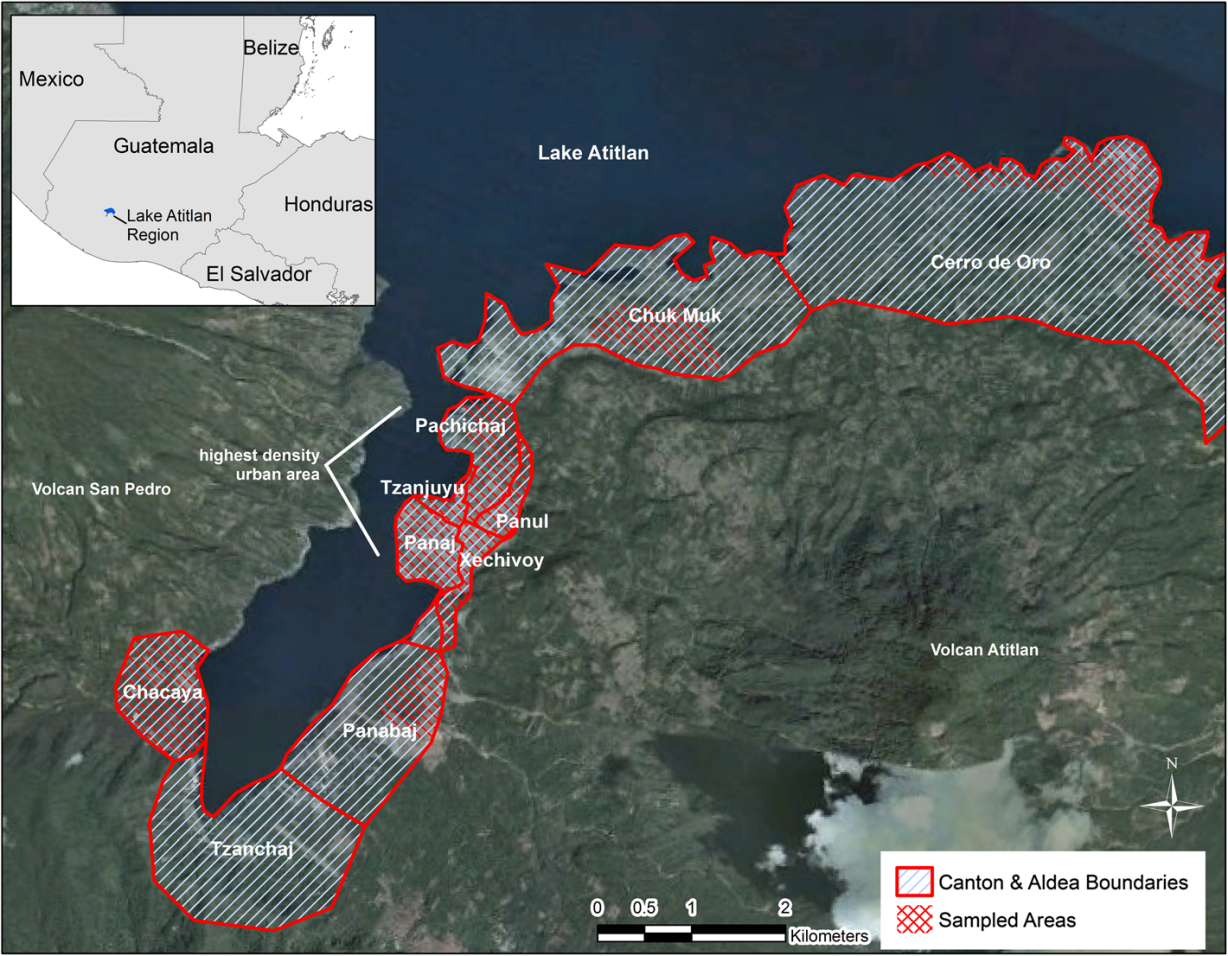
**Cantones and aldeas of Santiago Atitlán, Guatemala.** Note: Blue uni-directional hatch represents canton and aldea boundaries. Red hatch represents sampled areas.

The city is geographically divided into ten districts, called either cantones or aldeas (see Figure 1), which comprise approximately 22 square kilometers. Five of these are designated as urban cantones and are located closer to the city’s center: Xechivoy, Panaj, Tzanjuyu, Pachichaj and Panul. Two aldeas slightly less proximate to the city center are Chacaya and Cerro de Oro. The City also has three rural aldeas or cantones, located about 10 to 15 minutes from the town center by vehicle: Panabaj, Tzanchaj, and Chuk Muk.

Development patterns within and between each canton vary. Table 1 shows population numbers by canton, and population densities ranging from 1,230 to 37,015 people per square kilometer. Cantones in the town center contain single-, two-, and multi-story dwellings, main streets are paved, while walkways between housing structures, or viviendas, are often unpaved. Cantones outside of the city center have rural development patterns, with small clusters of primarily single-story dwellings (along roads), interspersed with agricultural land or forested areas. Streets in these areas are largely unpaved. Mountainous areas, moving away from the lakeshore are characterized by dense forest on steep slopes. In addition, this region (in particular the lake shore) is a destination second-home location for affluent foreigners. In more remote locations shoreline away from the town center, homes belonging to this population often are walled and gated for security purposes.

**Table 1:** Canton and aldea population density

**Canton/Aldea**	**Population**	**Area (km**^**2**^**)**	**Population density**^**a**^
Tzanjuyu	4,671	0.13	37,015
Panul	4,781	0.22	21,608
Panaj	8,269	0.50	16,591
Xechivoy	4,456	0.29	15,397
Pachichaj	4,681	0.55	8,493
Chuk Muk	4,000	0.59	6,736
Panabaj	2,294	0.43	5,382
Cerro de Oro	3,395	2.01	1,690
Chacaya	1,212	0.99	1,230

^a^persons per square kilometer.

The Guatemala Health Initiative (GHI) was founded in 2005 as a partnership between the University of Pennsylvania and the community of Santiago Atitlán. At the time of the opening of a new hospital (Hospitalito Atitlán) in 2005, University of Pennsylvania students performed a community health survey. This survey served as the epidemiological base for developing a Municipal Health Network of Santiago Atitlán involving the Municipal Centros de Salud and all other health organizations in the community. In 2012, community leaders in Santiago Atitlán worked with University of Pennsylvania faculty to update the community health survey to identify how needs have evolved over time. Results from the survey are to be published separately.

### Random spatial sampling

The research team developed a simple stratified random sampling methodology and used it to carry out a health survey between the months of June and July of 2012. This project is part of an ongoing protocol, approved by the Institutional Review Board at the University of Pennsylvania. The researchers also received ethical and social appropriateness review and approval from the municipality of Santiago Atitlán, the ministry of health Centro de Salud of Santiago Atitlán, and the Hospitalito Atitlán prior to beginning the study. Four undergraduate students from the University of Pennsylvania, for whom Spanish was not a native language, served as surveyors on this project. Eight translators, native to the Santiago Atitlán area and fluent in both Spanish and Tz’utujil, the local Mayan language, assisted with survey administration.

Cantones and aldeas of Santiago Atitlán served as the sampling frame. However, GIS and GPS technology had not been extensively used in the area previously, so GIS data on municipal boundaries did not exist prior to the start of the survey.^a^ Therefore the research team digitized an official map depicting canton and aldea boundaries. In addition, the research team toured the area on foot with a guide knowledgeable about boundary locations and a GPS unit (Garmin GPSMAP 62 s). Boundaries were compiled using ArcGIS software (ESRI, Inc.)^b^.

We sought to sample 1 in 20 households in Santiago Atitlán, stratified by canton or aldea district boundaries (for a 95% confidence level, and confidence interval of 5.7). Since maps of household locations did not exist, we created a stratified random spatial sample (using the random point generator tool) of x-y-coordinates in each canton or aldea. We excluded all known non-residential uses from the sampling area, for example water and cemetery. Due to the geography of the region, boundaries between land uses are striking and can be discerned from satellite image, e.g. water boundaries, and boundaries between residential areas and forest. The sampling size was determined based on resource and security constraints; the research team sought to work with available resources (four surveyors, eight-week survey duration, and two GPS units), and to avoid areas commonly known to be prone to armed-thefts. Assuming a 20% rate of failure to successfully recruit participants, or “fail-rate”, we generated sample points for 0.07% of the population for all cantones and aldeas (except the Tzanchaj aldea due to security concerns).

### Fieldwork

Prior to the study, surveyors were trained in the use of GPS units to 1) find pre-determined sample points in the field, and 2) “mark” or record the x-y-coordinates of the actual survey locations and locations of interest to the study (see appendix). Surveyors were instructed on how to download “waypoints”, or pre-determined x-y-coordinates for surveying onto GPS units (Additional file 1). Satellite images for use in the Garmin units and in the desktop software were obtained from Garmin’s BirdsEye satellite imagery service.

Surveyors always travelled in pairs to the field for security purposes. For each canton or aldea, surveyor teams made an initial trip or trips to the sample points using the GPS units. Surveyors would travel by “tuc tuc” (three-wheeled vehicle) to the canton or aldea vicinity and then by foot to sample points. At each sample point surveyors recorded in their notes a physical description of the household and its location. If the point was located away from a structure, such as in a field or forested area, surveyors were instructed to locate the nearest residence (within 1000 feet) to the right of the point when facing north. Surveyors were able to locate between 35 and 45 sample points in one day.

On the following day, surveyors returned to the field to conduct surveys at each of these points. Each of the four surveyors travelled with a translator. While the team had originally planned to do an initial visit with households to determine whether a translator was needed, they found that it was necessary to travel with translators full-time for a number of reasons: 1) Tz’utujil is the primary language spoken in this area, while approximately 50% of the population speak Spanish fluently (usually in addition to Tz’utujil in the case of native-born residents). The research team found that participants that indicated they could speak Spanish often were not completely fluent, and were unfamiliar with certain topics (described in Spanish) contained in the survey; 2) Translators helped improve access to respondents; 3) Scheduling a return visit required coordinating the respondents’, translators’ and surveyors’ schedules, and would have required more time than could be afforded; and 4) Translators held “local knowledge” helpful to surveyors in terms of navigating the field, communicating with and being respectful to participants. For example, in more rural areas where houses were detached and disperse, translators aided the recruitment process because they were helpful in identifying which buildings on-the-ground were residences.

Once surveyors returned to the residence, they sought-out a household member, 18 years of age or older, that held some familiarity with the health of other household members. Household members were not selected using a randomization process because there was often not more than one person present that met these criteria. If a qualified household member was not present, surveyors moved to the right (when facing the front door) until a residence with a qualified survey participant was found. If no household could be found for that point, due to time and resource constraints, the survey team would move on to the next point and would not return. This is the reason that a higher number of sample points were generated (0.02% higher than our targeted sample number). We define successful recruitment to be instances when surveyors successfully completed a survey for a pre-defined sample point. After the survey, the team compensated participants with eggs and at times corn, both valued commodities in the region.

We derived lessons learned from carrying out this methodology from participant observation during time spent on-the-ground with surveyors, as well as from informal unrecorded interviews with four surveyors during and after surveying. Interviews focused on the process of surveying, challenges experienced in each canton, and experiences related to the use of sampling technology.

## Results

### Survey preparation

This method introduces opportunities for efficient sampling in remote locations with limited population data. Yet, one time-consuming step involved with this method is the preparation of digital boundary data within which a random sample of points can be generated. Unlike cluster sampling methods that use grid placement, a random spatial sampling method requires that the boundaries of these geographies be specifically defined [8]. It is most desirable to use clusters for which population or census data are available.

Geographies in general can be classified as physical, social or administrative. Physical geographies represent areas of development such as a town with a distinct urban/forest or urban/agriculture boundary. In this case, researchers may walk or drive the periphery of the developed area with a GPS unit to delineate this physical boundary. Social geographies might include neighborhoods within urban areas. This type of boundary is likely to be most difficult to delineate, as ideas about the locations of boundaries and the names and identities of neighborhoods may be multiple and contested. When working with this type of geography, researchers must obtain multiple perspectives on naming and boundaries. It is also less likely that population data are available for socially-defined geographies such as neighborhoods, particularly when those neighborhoods are not bounded by streets with names or other fixed geographic markers. Geographies can also be administrative in nature, such as districts within municipal areas, and in this case it may be necessary to seek information on the location of these boundaries from official documents, maps, and local officials. In any case, creating spatial data sets representing these boundaries requires knowledge and skill using GPS units, spatial data, and GIS.

In the case of Santiago Atitlán, geographies representing administrative units are called cantones and aldeas, and are defined by the municipal government for social organization including census taking, resource distribution, and emergency management planning purposes. One challenge facing the research team in Santiago Atitlán was that canton and aldea boundaries were poorly documented within existing local municipality maps. It was important to delineate boundaries according to municipal understandings because census figures for each canton or aldea were used to calculate sample size. The most credible written source was a conceptual-level map showing rough canton and aldea boundaries created by the municipality. It was difficult to decipher the geographic locations of boundaries using this map; boundaries did not always correspond with streets, and those that did were not always clear because streets were not named. Second, canton and aldea names and boundaries were also socially defined.

There was a broad oral knowledge, especially among older residents, of aldea and canton boundaries that were defined by streets, structures, and natural objects such as rocks, streams, or a change in terrain. The research team carried a GPS unit on foot with a long-time resident knowledgeable about boundary locations. However, in the field, surveyors found a lack of agreement on canton or neighborhood identity, especially in boundary areas. We found that informal neighborhood identities were interspersed with official canton identities. Ultimately, the research team had to make calculated judgments about the locations of administrative, rather than social, boundaries.

### Technological limitations

One potential challenge in using the random spatial sampling method is availability of adequate technology and skill in its use. One significant technological challenge for this research team was the availability of adequate satellite imagery that could be used by both ArcMap (ESRI, Inc.), and the GPS units. Satellite images for use in the Garmin units and in the desktop software were obtained from Garmin’s BirdsEye satellite imagery service. BirdsEye satellite images of the Lake Atitlán area reflected on-the-ground conditions within the past two years. For example, the images reflected a large housing development located in a northern aldea, which was built after the 2005 hurricane and mudslides to house displaced residents. However, resolution of these images was not as high as was desired.

Google Earth provided satellite images of higher resolution (free of charge), and was used as a pre-navigation tool; viewing sample points in Google Earth before visiting the field helped orient the surveyors. However, Google Earth images were not as up to date as BirdsEye images; they reflected development as it existed prior to the 2005 hurricane, and were therefore of limited use in some areas. In addition, we found that the transportation layers provided by Google Earth, namely streets, were not correctly placed (they were displaced by up to 15 meters) based on our reconnaissance with GPS units. Finally, the lake itself had risen significantly in the years between both map making and the taking of different satellite images, which changed the land mass, covering some homes and other structures, and altering the shoreline canton definitions.

Another potential challenge, which we did not encounter, surrounds use of GPS units. In high conflict areas, use of GPS devices pose security, and therefore safety, concerns. However the Garmin units look similar to cell phones, which many residents own, and neither the public nor officials were suspect of GPS use. Surveyors found the GPS units to be durable and intuitive to use. They had relatively little difficulty using GPS units to navigate to sample points, or to mark actual survey locations. Effectively uploading newly marked points to personal computers, and to remote server for analysis, posed the most challenge.

### Household selection

While the random spatial sampling method potentially reduces bias associated with locating the cluster center and survey households in the spin-the-pen EPI method [9,10], there is still potential for introduction of surveyor bias in the household selection process at each survey point. In the random spatial sampling method, surveyors must follow a procedure to find the randomly generated points in the field and select the nearest household or group of households for surveying [5]. This process should be expected to vary by study and within studies depending on local conditions, such as density, cultural norms, available resources, surveyor familiarity with the local environment, and security concerns. Few studies provide detailed accounts of their methods, and retrospective feedback from surveyors, for this household selection process.

The research team was challenged to develop a protocol that would work in all cantones and aldeas within the study, which ranged in density and socio-demographic characteristics. The team successfully surveyed 56% to 84% of sample points by canton (see Table 2).

**Table 2:** Survey completion rates

**Canton/Aldea**	**Population density**^**a**^	**Sample number**	**Surveys completed**	**Percent completed**
Tzanjuyu	37,015	39	29	74%
Panul	21,608	38	28	74%
Panaj	16,591	69	58	84%
Xechivoy	15,397	37	21	57%
Pachichaj	8,493	39	22	56%
Chuk Muk	6,736	27	21	78%
Panabaj	5,382	19	13	68%
Cerro de Oro	1,690	24	19	79%
Chacaya	1,230	8	5	63%
**Total**		**300**	**216**	**72%**

^a^persons per square kilometer.

Settlement density presented challenges in the household selection process. In some cases, randomly selected points did not fall on a household, but instead on a non-residential building or an open area (such as forest, field or water). In semi-rural and rural areas, the likelihood of this occurrence was higher. In this instance, it was not possible to expect that randomly generated points would always coincide with household locations, and moving random points to coincide with households would remove the random nature of the sample. In this case, Kolbe et al. [4,17] located all residences within 20 yards or meters of the survey point, assigned these residences a number, and randomly selected a number in order to select the surveying location. When no households existed within this radius, another household was located at another random location using the same method. Grais et al. [8] located the closest housing cluster or compound to the right of the GPS point (when facing north). This study used a combination of these methods; surveyors located the closest household (within 1000 feet), to the east of the GPS point. If the GPS point fell on a non-residential building or on a household that was absent or declined participation, the research team moved to the household to the right of that building.

According to the surveyors, point location and household selection were the “easiest” in areas with higher density or easily navigable settlement pattern (such as the new development in Chuk Muk). While surveyors reported that surveying was most challenging in low-density areas, this did not equate to low completion rates in low-density aldeas such as Cerro de Oro and Chuk Muk. Instead, we found low completion rates to be associated with either low density, abandoned homes, or security concerns.

Surveyors also reported that it was difficult to survey in cantones most populated by temporary residents and non-residential uses, such as Xechivoy. Common in some areas of the Santiago Atitlán area (such as the lakeshore) are temporary residences of foreigners. In many cases the survey team found these homes to be uninhabited, and walled or gated. The walled compounds also presented physical barriers to accessing points beyond. In all of these challenging cases, surveyors reported that translators were helpful in finding residential buildings nearest to the sample point.

Although low settlement density reduced successful survey rates for our research team, successful recruitment was high (above 78%) in two lower-density areas (Chuk Muk canton and Cerro de Oro aldea). Surveyors attributed the higher rate in Chuk Muk to the fact that residential development in this area is new (built after the 2005 hurricane) and therefore easy to navigate. In Cerro de Oro, surveyors attributed the relatively high success rate to development patterns that were easy to navigate, and the skills and help of the translator.

## Discussion and conclusions

Studies are emerging that use this combination of technologies to develop a random spatial sample, avoiding potential biases common in two-stage cluster sample approaches. This study describes an accessible stratified random sampling method combining GIS software, GPS technology and up-to-date satellite imagery, for surveying in remote regions with limited resources, infrastructure, and demographic data. We offer a more detailed exploration of methodological process and concerns, specifically regarding survey preparation, technological limitations and in-the-field household selection.

While this method provides opportunity for more efficient sampling compared to traditional methods, it involves time-intensive work primarily in preparing the random sample; digitizing or delineating cluster boundaries when electronic boundary files do not exist. The availability of population or household counts within clusters allows generation of sample points consistent with population density. Delineation methods must be determined depending on the shape and nature of the cluster (whether they are physical, social or administrative) and availability of information. The research team encountered most difficulty in this study due to lack of definitive maps and information about the specific boundaries of cantones, which had both administrative and social characteristics. One limitation of this method, similar to the EPI method, is that it relies on the existence of up-to-date household or population counts by district/cluster.

Based on our experience, researchers may also face technological challenges associated with proprietary issues between spatial software and hardware, namely in the use of consistent satellite imagery, between spatial software (ESRI’s ArcMap), and the GPS units (Garmin).

While our method offers a potential way to avoid biases inherent in the EPI spin-the-pen method, namely the favoring of households located near the center of grid clusters, surveyor judgment is still involved in household selection with this method and care must be taken to avoid systematic biases. Household selection processes must be adapted to varying development typologies, housing densities, and target respondents. While Grais [8] found that a similar method favors (leads to higher survey-completion rate) household selection in rural areas, to the contrary our method favored more urban areas (between 16,000 to 37,000 persons per square kilometer), and new residential developments.

Geographically sampled household surveys provide important information on health indicators. This study describes an accessible geographic random sampling method that provides a route to avoid potential biases inherent in two-stage cluster sampling methods in remote rural regions. It also offers a candid description of on-the-ground challenges faced by surveyors. Although this method should be further validated and compared with more established methods to determine its utility in social survey applications, it shows promise for use in developing nations and resource-challenged environments where detailed geographic and human census data are less available.

## Endnotes

^a^The Guatemala Instituto Nacional de Estadistica performs a household census approximately every decade. At the time of the study, the most recent census had been completed over 10 years prior (2002), and maps were in paper-format and had not been georeferenced. The research team was not able to obtain these maps prior to designing the sampling frame. Future studies should make use of such resources.

^b^At the time of the study, the GPS unit (Garmin, Inc.) cost approximately $280 USD, and ArcGIS (ESRI, Inc.) cost $1,500 for an individual site-license. Other project costs were associated with travel and labor of translators (approximately 500 labor hours).

## Competing interests

The authors declare that they have no competing interests.

## Authors’ contributions

MK led development of the study design, creation of random sample, delineation of canton/aldea boundaries, training and interviewing of surveyors, and writing of manuscript. KB contributed to study concept and acquisition of data including co-management of the research program, reviewing and revising the manuscript, and final approval with the other authors. FB contributed to the study design, and reviewing and revising the manuscript. CB contributed to the study design, and reviewing and revising the manuscript. All authors read and approved the final manuscript.

## Pre-publication history

The pre-publication history for this paper can be accessed here:

http://www.biomedcentral.com/1471-2458/14/338/prepub

## Supplementary Material

Additional file 1 GPS Training Guide. Click here for file
