# Supplementary material for: A random spatial sampling method in a rural developing nation
Source: BMC Public Health. 2014 Apr 10;14:338. doi: 10.1186/1471-2458-14-338 (PMC4021077; doi:10.1186/1471-2458-14-338)
Supplement: Additional file 1 — GPS Training Guide. [file 1471-2458-14-338-S1.pdf]

Guatemala Health Initiative
Summer 2012
Introduction to GPS Field Exercise

Objectives:

The overall objective of this exercise is to allow you to obtain hands-on experience with a GPS receiver, so that you may use it to support your research in Santiago Atitlan this summer. You will learn how to:

- locate your position & navigate through a user-interface,
- navigate to a predetermined waypoint. This skill may be useful in sampling for health survey respondents.
- mark a waypoint. This skill will be useful in recording the geographic location of your participants' homes or other places important in your research such as health clinics, for later spatial analysis; and
- use the BaseCamp software which allows you to save and edit your data on your computer.
- view your gps data in Google Maps and BaseCamp

Hints and trouble-shooting:

- Don't worry if your location readings "jump around" a bit, small fluctuations are normal. If you're concerned about fluctuations, move further away from buildings or trees.
- Going in the order the points are listed is the fastest route.

Part 1: Introduction to Garmin GPSMAP 62S

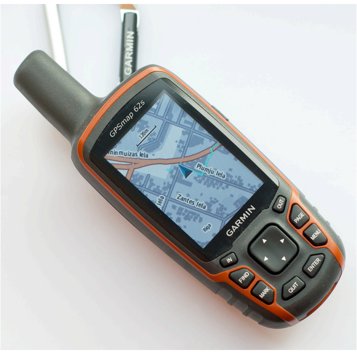

Step 1: Satellite Position

a. Go outside to an open area and turn on the device.
b. Wait while the device searches for satellites. A question mark flashes while the device determines your location. It may take a few minutes to acquire signals. Some strategies to get the receiver to locate satellite signals, and your location:
   2. Stay away from buildings and large trees.
   3. Hold the receiver away from yourself, or even set it down a few feet away from you.
c. To check status, and control backlight for the screen, quickly press the power button. The GPS bars indicate satellite strength; all bars green means it has acquired signals.

Step 2: Navigate to pre-selected locations (waypoints)
It's important to use a sampling method that will allow you to overcome potential bias in the results from surveys, such as your health survey. We will use a variation of areal sampling, or cluster sampling, to reduce biases that might come from a convenience method (e.g. surveying homes near us, or using social networks to find new participants). Geographic Information Software can help to select random points within a geographic area, such as the city limits of Santiago Atitlan, or within each canton. I will help to create these random points, and you will have these points in the gps when you travel to Guatemala. Once in the field, you'll need to know how to use the gps to find these points. The following is an exercise to mimic this task.

a. Push the ***MENU*** button (may need to push it twice) to go to the *Main Menu*.

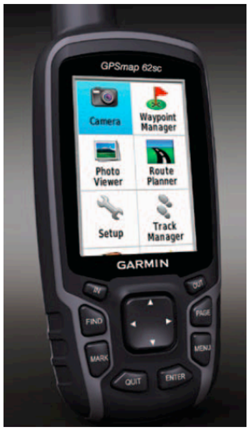

b. Select *Waypoint Manager*

c. See *Waypoints* listed in the dialog box. ****Please note that Group 1 should find waypoints 1-1 through 1-10, and Group 2 should locate waypoints 2-1 through 2-9. Each participant should have a chance to navigate to at least 2 waypoints.**

d. Highlight and select (using the arrows and *ENTER* button) the waypoint assigned to your group. To see the waypoint with the map in the background, scroll down using the arrows and select the *Map* option.

e. Please keep in mind that navigating on Penn's campus will be more simple than navigating streets and paths in Santiago.

f. Navigate to all waypoints and fill in the following table (hint: you should find two types of objects, smaller than buildings):

Waypoint ID	Object

Step 3: Creating Waypoints or Spatial IDs for field locations.
The ability to record the spatial location (x-y-coordinates) of your survey respondents' homes, and other places important in your research such as health clinics, will allow you to do a spatial analysis of your results. What type of spatial questions might this information assist in answering?

This exercise will help you mimic your needs assessment or diabetes survey in Santiago. For this field exercise, you are to mark the locations of at least 10 food trucks. The goal of your survey is to find out the type of food, and the availability of sugary soda. You can later use this information to decide where to go, or analyze proximity of sugary soda availability to places where children spend time (among other possibilities).

a. Find a food truck
b. Press the *Mark* button. When the *Mark Waypoint* screen appears highlight the number and select it. Notice that the device stores all waypoints in one folder. In order to distinguish waypoints associated with your study with other waypoints, you will need to use your own ID system. Rename the waypoint, e.g. **fts_01** ("fts" could be short for food-truck study).
c. Highlight the blue flag above the waypoint name and select an appropriate icon that represents the waypoint. You may want to use the marker to indicate the availability of sugary soda.
d. In the Notes field, enter the answers to your survey questions (i.e. food type, availability of sugary soda). e.g.
   Notes:
   Chinese
   Yes

Also record your ID numbers and survey answers:

Waypoint ID	Food Type	Sugary Soda?

Part 2: Data Management and Viewing

Step 4: Editing and Deleting Waypoints

You may make mistakes when creating waypoints, and need to either edit or delete waypoints at sometime.

a. To edit a waypoint, from the main menu select Waypoint Manager. Select a waypoint, select an attribute and edit, then select Done.
b. To delete a waypoint, from the main menu select Waypoint Manager, select a waypoint, press the MENU button and select Delete, Yes. Be careful not to delete all Waypoints which is a possibility if you hit the MENU button before selecting a waypoint.

Step 5: Viewing and Analyzing Points using Google Earth and ArcGIS

a. To simply view your points using Google Earth, connect the device via mini usb adaptor to your laptop. Find your waypoints in the "GPX" folder of the Garmin folder. Open Google Earth and open this .gpx file.

b. In order to manage your waypoints, tracks and routes and satellite images, you need to use Garmin's Basecamp software. Download the software here:

   http://www.garmin.com/us/products/onthetrail/basecamp

Other potentially useful notes:

a. Your gps device keeps track of your location whenever it is on; it creates tracks of your route. It may be useful to see this route, in the event that you get lost or want to remember where you have been. Right now, the tracks are recording but not displaying on the devices. To change this, go to Settings, Tracks, highlight the Track Log and hit Enter, then select "Record, Show on Map"
b. When you are searching for a waypoint, the gps has a feature to help you navigate there in the form of a pink line from your location to the point. At times this feature is a distraction. To turn this off, press the Find button and select "Stop Navigation"
c. To download new satellite images, open Basecamp, connect the gps device. Highlight My Collection, then go to Utilities, Download Satellite Imagery from BirdsEye. Use the rectangle tool to draw a rectangle around the area you'd like to download.
